# Three-Dimensional Printed Hydroxyapatite Bone Substitutes Designed by a Novel Periodic Minimal Surface Algorithm Are Highly Osteoconductive

**DOI:** 10.1089/3dp.2022.0134

**Published:** 2023-10-10

**Authors:** Ekaterina Maevskaia, Nupur Khera, Chafik Ghayor, Indranil Bhattacharya, Julien Guerrero, Flora Nicholls, Christian Waldvogel, Ralph Bärtschi, Lea Fritschi, Dániel Salamon, Mutlu Özcan, Patrick Malgaroli, Daniel Seiler, Michael de Wild, Franz E. Weber

**Affiliations:** ^1^ Oral Biotechnology & Bioengineering, Center of Dental Medicine, University of Zurich, Zurich, Switzerland.; ^2^ Central Biological Laboratory, University Hospital Zurich, Zurich, Switzerland.; ^3^ Spherene, Zurich, Switzerland.; ^4^ Center of Dental Medicine, Division of Dental Biomaterials, Clinic for Reconstructive Dentistry, University of Zurich, Zurich, Switzerland.; ^5^ Institute for Medical Engineering and Medical Informatics IM2, School of Life Sciences, University of Applied Sciences Northwestern Switzerland, FHNW, Muttenz, Switzerland.; ^6^ CABMM, Center for Applied Biotechnology and Molecular Medicine, University of Zurich, Zurich, Switzerland.

**Keywords:** triply periodic minimal surface, TPMS, adaptive density minimal surfaces, ADMS, hydroxyapatite, osteoconduction, microarchitecture, bone substitute, additive manufacturing, 3D printing, ceramics, titanium

## Abstract

Autologous bone remains the gold standard bone substitute in clinical practice. Therefore, the microarchitecture of newly developed synthetic bone substitutes, which reflects the spatial distribution of materials in the scaffold, aims to recapitulate the natural bone microarchitecture. However, the natural bone microarchitecture is optimized to obtain a mechanically stable, lightweight structure adapted to the biomechanical loading situation. In the context of synthetic bone substitutes, the application of a Triply Periodic Minimum Surface (TPMS) algorithm can yield stable lightweight microarchitectures that, despite their demanding architectural complexity, can be produced by additive manufacturing. In this study, we applied the TPMS derivative Adaptive Density Minimal Surfaces (ADMS) algorithm to produce scaffolds from hydroxyapatite (HA) using a lithography-based layer-by-layer methodology and compared them with an established highly osteoconductive lattice microarchitecture. We characterized them for compression strength, osteoconductivity, and bone regeneration. The *in vivo* results, based on a rabbit calvaria defect model, showed that bony ingrowth into ADMS constructs as a measure of osteoconduction depended on minimal constriction as it limited the maximum apparent pore diameter in these scaffolds to 1.53 mm. Osteoconduction decreased significantly at a diameter of 1.76 mm. The most suitable ADMS microarchitecture was as osteoconductive as a highly osteoconductive orthogonal lattice microarchitecture in noncritical- and critical-size calvarial defects. However, the compression strength and microarchitectural integrity *in vivo* were significantly higher for scaffolds with their microarchitecture based on the ADMS algorithm when compared with high-connectivity lattice microarchitectures. Therefore, bone substitutes with high osteoconductivity can be designed with the advantages of the ADMS-based microarchitectures. As TPMS and ADMS microarchitectures are true lightweight structures optimized for high mechanical stability with a minimal amount of material, such microarchitectures appear most suitable for bone substitutes used in clinical settings to treat bone defects in weight-bearing and non-weight-bearing sites.


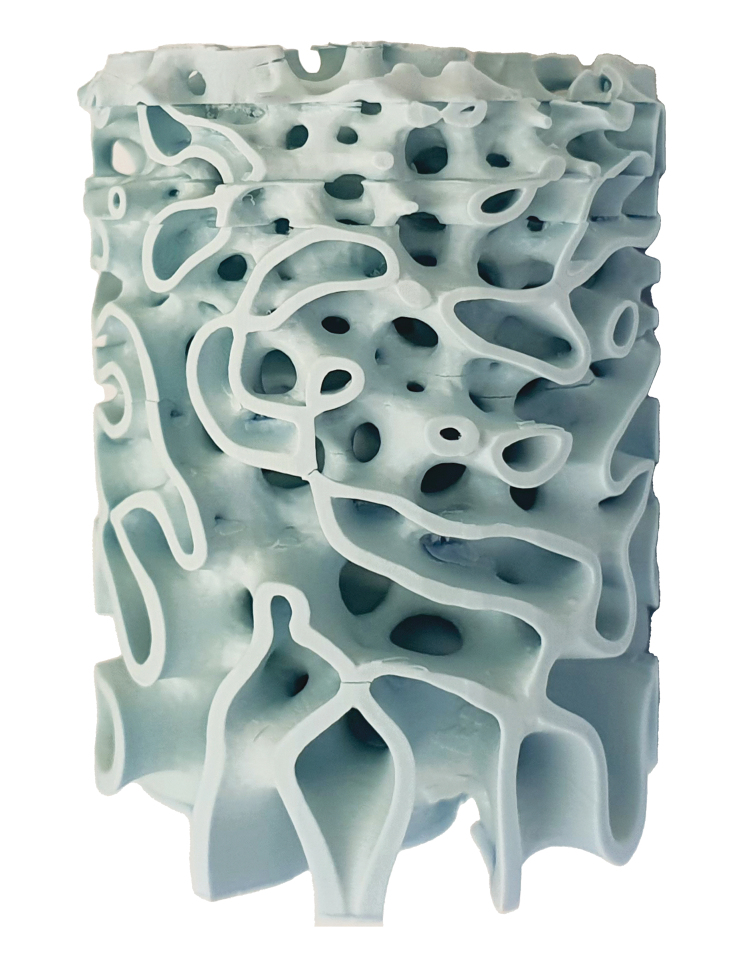


## Introduction

Bone is a lightweight composite material because it combines high rigidity and strength relative to its weight. The overall bone architecture is composed of an outer shell made of dense tissue known as cortical bone that surrounds cancellous bone and a network of the interconnected plate- and rod-like struts called trabeculae (∼50–300 μm in thickness).^1^ According to Wolff´s law,^2^ healthy bone will constantly adjust to the locally applied load with constant bone turnover. Therefore, due to this remodeling, trabeculae in cancellous bone are preferentially aligned in the direction of the biomechanical stresses generated by physical activity. In the event of a bony defect, when autologous bone is transplanted to bridge it, the trabecular network of this transplant represents a porous microarchitecture where osteoconduction can occur.

Osteoconduction is defined as the ingrowth of sprouting capillaries, perivascular tissue, and osteoprogenitor cells from a bony bed into the three-dimensional (3D) structure of a porous implant (adapted from Cornell and Lane^3,4^) used as a guiding cue to bridge a defect in bony tissue.^5^ In bone tissue engineering, as a combination of scaffolds, cells, and growth factors,^6^ osteoconduction is heavily dependent on the scaffold microarchitecture.^5^ Porosity was the focus of scaffold development during the advent of the bone tissue engineering era.^7^ However, the main methodology depends on water-soluble porogens in the size of the preferred pore dimension.^8^ After curing the scaffold material, the porogens were leached out, leaving behind a stochastic pore distribution and pore system.^9^ The pore size in such a scaffold is generally approximated by the diameter of the largest sphere that can be fitted into the pore system based on ground sections.^10^ The constriction or bottleneck of such a pore system is the diameter of the sphere that can be fitted to the smallest connection in the pore system.

The recent production revolution of layer-by-layer 3D printing technology facilitates the realization of the demanding, predefined pore systems such that the microarchitectures, pore dimensions, and constrictions in bone substitutes are defined and designed beforehand and are ideally optimized for osteoconduction and bone regeneration.^11–13^ The most widely used 3D printing technology is based on extruding filaments by melting the material,^14^ a solution/slurry/gel filament,^15,16^ or fused deposition.^17,18^ Since filament-based 3D printing constrains possible variations in microarchitectures, more demanding microarchitectures can only be realized by stereolithography, selective laser sintering,^19^ or 3D printing on a powder bed.^20^ One complex microarchitecture family with the potential to become a standard in bone tissue engineering is based on triply periodic minimum surface (TPMS) algorithms.^21–23^ These algorithms describe lightweight microarchitectures associated with high strength.

The TPMS algorithms evolved from the mathematical problem of finding the surface that formed the smallest area for a given perimeter. This problem for a surface formed between four points located on one of the four sides of a tetrahedron was solved by Schwarz in 1890^24^ and led to the diamond (D) and primitive (P) surfaces. In 1970, Schoen introduced the gyroid (G) surface, another minimal surface^25^ with the property that the mean curvature vanishes at every location on the surface and becomes zero. The common features of all TPMS surfaces include their periodicity in three independent directions (hence “triply periodic minimal surface”), extending infinitely, devoid of self-intersections, and partitioning the space into two independent labyrinths.^22^ After the G-surface was identified at the interface between the inorganic crystalline and organic amorphous matter in the echinoderm plates,^26^ the D-, P-, and G-surfaces were recognized in all the kingdoms of living organisms.^22^ For example, in plants grown in the dark, the lamellar structure of chloroplasts adopts P-surface topology and symmetry.^27^

During the skin barrier formation in humans, a “TPMS-like lamellar membrane” transition occurs inside the tubuloreticular cisternal membrane system of upper granular cells.^28^ Although the trabecular structure of the bone where the support structures bear predefined loads resembles a gothic cathedral,^29^ the similarity between the TPMS and the overall bone structure is based on the common lightweight characteristics and not on an algorithm (TPMS or adaptive density minimal surfaces [ADMS]). This notion is supported by the finding that unlike the basic element of TPMS structures, the mean curvature of any point on the surface of the cancellous bone diverts from zero and is found to be negative, demonstrating that their surfaces are hyperbolic on average.^30^

In bone microarchitecture, “minimal surface” is associated with lightweight and high-strength characteristics combined with low mass. Moreover, the average curvature of any point on the surface is zero, leading to a uniform stress distribution under load and high strength. Owing to this combination, TPMS microarchitectures appear attractive for designing bone substitutes. Some reports of their use in bone tissue engineering have already been published.^31–34^ However, reports of *in vivo* applications of TPMS structures in bone substitutes are still scarce and lack direct comparisons to highly osteoconductive microarchitectures.^21,35^

In TPMS structures, structural thickness and orientation distribution are constant, leading to a directional dependency of the mechanical properties.^36^ Low anisotropy would be desirable for bone substitutes to be placed at any site. Therefore, we applied the novel ADMS microarchitecture characterized by a lack of spikes in the distribution of the orientation of their surface and an equal mechanical performance in all directions, which can be adjusted to satisfy a given load case. Similar to TPMS structures, ADMS are doubly curved at each point, such that the mean curvature is zero at every point. The main objective of this project was to produce bone substitutes from hydroxyapatite (HA) by lithography-based 3D printing with ADMS structures of three different pore system constrictions and a reference ADMS structure from titanium (Ti) by Selective Laser Melting (SLM) to identify the most osteoconductive ADMS-based microarchitecture and compare it *in vivo* to a highly osteoconductive, well-interconnected lattice microarchitecture that is already well documented.^37^

## Materials and Methods

### Implant production

HA-based scaffolds were produced with HA slurry LithaBone™ HA 400 (Lithoz, Vienna, Austria) using a CeraFab7500 from Lithoz, as previously reported.^38^ The ADMS scaffolds were characterized by a minimum constriction of 0.5, 0.8, or 1.1 mm and a wall thickness of 0.2 mm. As a control, we used a lattice microarchitecture known for high osteoconductivity with struts of 0.3 mm at a distance of 0.8 mm.^37^ The maximum dwelling time was set to 1300°C to produce mechanically stable osteoconductive implants.^38^ Spherene AG (Zurich, Switzerland) provided standard triangular language (STL) files describing the micro- and macroarchitecture of the scaffolds. The ADMS and lattice microarchitecture filled a 6.0-mm-diameter and 4.5-mm-high cylinder. The overall construct contained two additional rings as follows: (1) To prevent immersion of the implant into the bone defect beyond 2.5 mm, a solid ring of 1.0 mm thickness encased the upper 2.0 mm of the cylinders with ADMS or lattice microarchitecture. (2) The design included a solid ring with an outer diameter of 6.0 mm, a height of 0.5 mm, and 0.3 mm thick was included in the design.

For critical-size defects, the cylinders with the ADMS and lattice microarchitecture were 4.5 mm in height and 15.0 mm in diameter to stabilize the lower part of the cylinder. The two rings for stabilization and prevention of immersion were designed according to the scaffolds for noncritical-size defects. A solid ring 1.0 mm thick encased the upper 2.0 mm of the cylinders, and a solid ring with an outer diameter of 15.0 mm, a height of 0.5 mm, and 0.3 mm thick stabilized the lower part with ADMS or lattice microarchitecture. Upon producing the green bodies, the sintered scaffolds were transferred from the oven to a sterile bench in containers, packed for transportation into the operation workflow, and used as bone substitute implants without further sterilization.

For the additive manufacturing of the titanium-based scaffolds, the same STL files of the ADMS models were imported into Magics software (V21; Materialise) where the support structures to the building platform were created using “block type” support. Note that no supports were necessary within the ADMS structures. The manufacturing of the samples was done using a SLM 250 HL system (by SLM Solutions GmbH, Lübeck, Germany) with a building platform of 250 × 250 mm^2^, and with integrated powder reconditioning and sieving unit. The SLM system used a continuous wave 200 W Ytterbium fiber laser with a wavelength between 1068 and 1095 nm. A layer height of 30 μm was used to print a commercially pure titanium powder (Ti grade II according to ASTM F67; SLM-Solutions GmbH) with a d50 of 41 ± 2 μm (Particle size analysis with Helos/KF + incl. RODOS/ + VIBRI particle size distribution analysis set up by SympaTec GmbH, Clausthal-Zellerfeld, Germany).

The printing laser parameters were set to 100 W nominal laser power for the outer contour at a scanning speed of 550 mm/s and 175 W for the inner contour with a scanning speed of 833 mm/s. After the SLM process, the parts were carefully detached manually from the building platform. The downward facing support structures were then broken off at perforated support breaking points.

### Implant characterization and microcomputed tomography scanning

Wall thickness and maximal diameter of the two-dimensional (2D)-sphere fitting in the microarchitecture were measured on the ground sections using image analysis software (Image-Pro Plus^®^; Media Cybernetic, Silver Springs, MD). Microcomputed tomography (microCT) scanning of the scaffolds was performed with a SKYSCAN 1272 (Bruker, Kontich, Belgium) with the following settings: voltage 90 kV (0.5 mm Al +0.038 mm Cu filter), current 111 μA, pixel size 10.9 μm, and rotation step 0.2°. The reconstruction was performed using NRecon software. The analysis of the scaffold morphology (volume, porosity, and surface area) determined from three samples of each microarchitecture was performed using the CTAn software. Bruker supplied all software required for the reconstruction and analysis. The region of interest was a cylinder with an ADMS or lattice structure without the outer rings.

### Compression strength measurements

The comparative compression strength was based on scaffolds designed for noncritical size, 6 mm cylinder defects, enlarged by the Cerafab7500 software by a factor of 1.5, in the *x*, *y*, and *z* directions. The specimens were mounted in the jig of a universal testing machine (Zwick ROELL Z2.5 MA 18-1-3/7, Ulm, Germany). The occlusal surface (63.62 mm^2^) in the direction of the building layers was subjected to compressive loading at a crosshead speed of 1 mm/minute. The stress–strain curve was analyzed using a software (TestXpert V11.02; Zwick ROELL) to determine the maximum compression strength.

### Surgical procedure

Osteoconductivity of scaffolds was assessed *in vivo* by inserting the four 6-mm calvaria defect models (500, 800, and 1100 μm ADMS or lattice microarchitecture scaffolds) and the Ti-ADMS 800 μm scaffolds into ten adult New Zealand white rabbits, as reported previously.^39^ In brief: “Animals were anesthetized by an injection of 65 mg/kg ketamine and 4 mg/kg xylazine. During operation, isoflurane/O_2_ was applied. After the disinfection, a straight incision from the nasal bone to the midsagittal crest was performed with a scalpel, the soft tissues reflected, and the periosteum removed. In the area of the right and left parietal and frontal bones, four evenly distributed 6-mm diameter craniotomy defects or one central 15-mm defect were marked with a trephine bur followed by the use of round burrs of different diameters starting from 6 to 1 mm to generate a full-thickness calvarial defect without damaging the dura. Finally, bone debris was removed by flushing the defects with sterile physiological saline solution.” For the 15-mm critical-size defect, which was used to compare the most suitable ADMS to the lattice microarchitecture, another 13 animals were operated on as previously described.^40^

Animals had unrestricted access to drinking water and were fed a standard laboratory diet. The weights of the animals ranged from 3.5 to 4.5 kg. The protocol was evaluated and approved by the local Ethics Committee (Veterinäramt Zürich, Zürich, Switzerland; 065/2018). All procedures were conducted in accordance with local guidelines. Four weeks after the operation with the 6-mm, noncritical-size defects and 8 weeks after the operation involving the 15-mm critical-size defects, the rabbits were anesthetized and sacrificed by an overdose of pentobarbital. The craniums containing the four or single craniotomy sites were removed and placed in 40% ethanol. Embedding was performed as previously reported.^40^

### Histomorphometry

A single ground section from the middle of each implant was evaluated using image analysis software (Image-Pro Plus; Media Cybernetic). The area of interest (AOI) was determined by the 6- or 15-mm defect width and the area fraction of the implant submerged into the bony defect. The regenerated area in the AOI was determined as the percentage of bone and bone integrated scaffolds in the AOI (bony area %).

### Bone bridging

Osteoconductivity was quantified by the extent of the bony-bridged defects, as previously reported.^41^ Briefly, bone tissue was identified by Toluidine Blue staining, and areas in the AOI were projected onto the *x*-axis. Next, all stretches of the *x*-axis with bone tissue were summed and related to the 6- and 15-mm defect widths, respectively. Bone bridging is given as a percentage of the 6- or 15-mm defect width where bone formation had occurred.

### Statistics

The primary analysis unit for *in vivo* experiments was a defect. Data from 9 to 10 rabbits for the 6-mm defect and 6 to 7 rabbits for the 15-mm defects were analyzed in each group. The Kruskal–Wallis test followed by pairwise comparison of treatment modalities with the Mann–Whitney test for independent data (IBM SPSS v.19) were applied. The Jonckheere–Terpstra test was used to determine statistical significance between groups of decreasing level of constriction. Statistical significance was set to 0.05 and the *p*-values are displayed in the graphs. If not specified, the *p*-values were determined by the Mann–Whitney test. Values are represented as mean ± standard deviation in the text or median ± lower/upper quartile in the graphs.

## Results

### Scaffold production

**FIG. 1.**
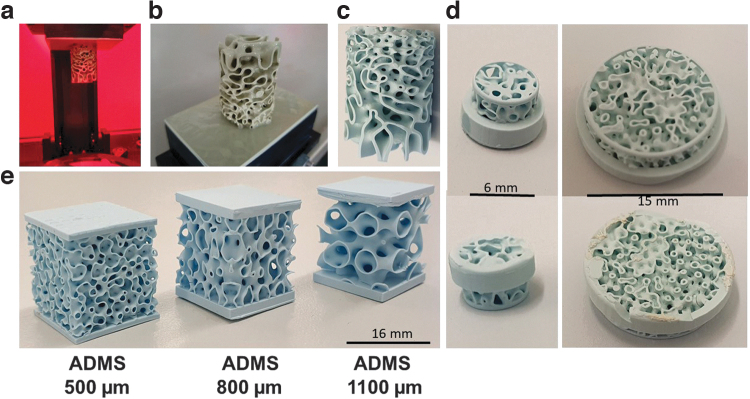
3D printing of ADMS structures. ADMS structure during printing in the CeraFab7500 machine **(a)**. ADMS structure after removal from the machine and attached to the building platform **(b)**. The same ADMS structure after sintering at 1300°C **(c)**. The diameter of the ADMS structure was 23 mm **(c)**. Six and fifteen millimeter scaffolds for animal testing are shown from the upper and lower sides **(d)**. Scale is provided. Diverse ADMS structures with minimal constriction diameter as listed **(e)**. Scale is provided. 3D, three-dimensional; ADMS, adaptive density minimal surface.

Scaffolds designed with the ADMS algorithm can be produced by the lithography-based layer-by-layer methodology or by SLM without a support structure (Fig. 1). After producing the noncritical-size defect scaffolds, the porosity and surface were determined from a cylinder 4.5 mm in height and 6 mm in diameter filled with the diverse microarchitectures by microCT and compared with the data encrypted in the STL file. The data compiled in Table 1 show that the porosity of HA-printed scaffolds determined by microCT was 3–8% lower than that encrypted in the STL file. Even more pronounced was the discrepancy between the STL file and printed scaffold in the surface area because, depending on the microarchitecture, the surface of the STL file *in silico* was 14–36% higher than measured in the printed HA-scaffolds by microCT. Minimum constriction or bottleneck between interconnected pores as preset in the STL file and chosen as the primary determinant of the three ADMS designs influenced the apparent 2D maximal sphere diameter reaching its maximum of 1.86 ± 0.17 mm in the ADMS 1100 μm design.

**Table 1.** Characteristics of Microarchitectures

Microarchitecture in HA	Porosity STL-file(%)	Porosity microCT (%)	Surface area microCT (mm^2^)	Surface area STL-file (mm^2^)	Minimal constriction in STL-file (mm)	2D maximal sphere diameter fitting in architecture (mm)
ADMS 500 μm	68.71	62.78 ± 1.87	376.06 ± 14.34	512.40	0.50	1.53 ± 0.12
ADMS 800 μm	71.16	64.57 ± 1.30	388.39 ± 43.19	481.90	0.80	1.51 ± 0.15
ADMS 1100 μm	78.36	71.55 ± 1.07	300.58 ± 22.28	381.97	1.10	1.86 ± 0.17
Lattice	79.40	75.58 ± 1.20	307.88 ± 32.03	378.60	0.80	1.07 ± 0.04

Comparison between STL file and microCT of HA-based scaffolds.

2D, two-dimensional; ADMS, adaptive density minimal surfaces; HA, hydroxyapatite; microCT, microcomputed tomography; STL, standard triangular language.

The material used for 3D printing and methodology affected the characteristics of the printed scaffolds, despite an identical STL file being used to program the printing systems (Table 2). For the ADMS 800 μm microarchitecture, the minimum and maximum wall thicknesses were higher if the scaffold was produced by a lithography-based methodology using a HA/binder-slurry compared with the production with a selective laser-melting methodology in a titanium powder bed. This significant increase in wall thickness increased the maximum diameter of a 2D-sphere fitting into the ground sections with ADMS 800 μm microarchitecture from 1.51 ± 0.16 mm for HA-scaffolds to 1.76 ± 0.12 mm for titanium scaffolds. Therefore, the central determinant of osteoconduction and bone regeneration of ADMS microarchitectures appears to be the diameter of the largest sphere that fits into the pore system.

**Table 2.** Adaptive Density Minimal Surfaces 800 μm Microarchitecture Characteristics and *In Vivo* Performance Dependent on Production Methodology and Material (Hydroxyapatite or Titanium)

ADMS 800 μm microarchitecture material	Wall thickness STL-file (mm)	Minimal wall thickness measured (mm)	Maximal wall thickness measured (mm)	2D maximal sphere diameter fitting in architecture (mm)	Bony bridging (%)	Bony regenerated area (%)	
HA	0.20	0.25 ± 0.03	0.31 ± 0.06	1.51 ± 0.16	82.85 ± 18.48	65.46 ± 42.20	
Ti	0.20	0.15 ± 0.02	0.28 ± 0.04	1.76 ± 0.12	50.94 ± 26.07	28.53 ± 25.60	

Ti, titanium.

### Mechanical testing of scaffolds

**FIG. 2.**
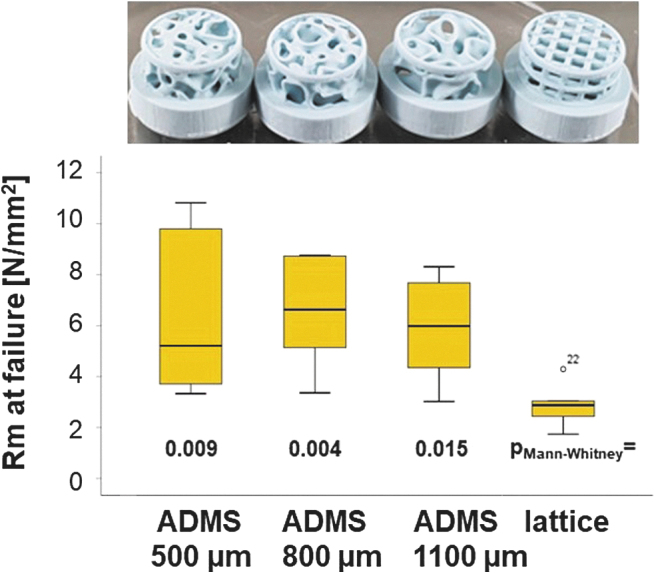
Compression strength of ADMS and lattice microarchitectures from HA. In the *upper panel*, all tested scaffold types are displayed in the same order as in the graph. The graph, in the lower panel visualizes the compression strength of all tested scaffold types, as listed, in a box-blot ranging from the 25th (lower quartile) to the 75th (upper quartile) percentile, with the median displayed as a *solid black line* and *whiskers* extending to the minimum and maximum values. Values outside the box blot are shown individually. HA, hydroxyapatite.

The compression strengths of ADMS 500, 800, and 1100 μm were evaluated and compared with the lattice architecture of samples derived from the STL file for scaffolds designed for the 6-mm defect model. To increase the initial stability of these scaffolds, the STL file of each 6-mm *in vivo* sample was transferred to the Cerafab7500 software and scaled up in *x*, *y*, and *z*-dimensions by a factor of 1.5, printed, and sintered. The resulting scaffolds (Fig. 2, upper panel) were subjected to compression analysis. The maximum compression strength of ADMS 500 μm was 6.34 ± 3.20 N/mm^2^; for ADMS 800 μm 6.54 ± 2.11 N/mm^2^; for ADMS 1100 μm 5.88 ± 1.98 N/mm^2^; and for orthogonal lattice 2.87 ± 0.84 N/mm^2^ (Fig. 2, lower panel). The lattice-based scaffold showed a significantly lower compression strength than all the tested ADMS structures.

### Osteoconductivity and bony regeneration capacity of ADMS and lattice-based scaffolds in noncritical-size defects *in vivo*

**FIG. 3.**
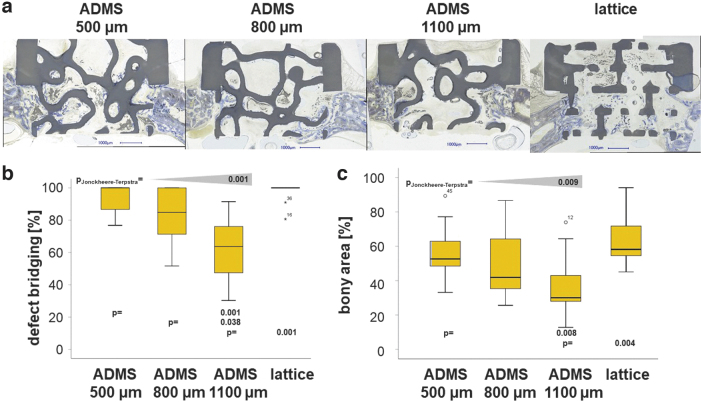
Osteoconduction and bone regeneration in ADMS and lattice microarchitectures. Histological sections from the middle of the noncritical-size defects were treated with ADMS- and lattice-derived scaffolds after 4 weeks of recovery **(a)**. Scale bars in *blue* indicate 1000 μm. Original magnifications were 100-fold. Bone appears as *grayish purple* to *purple*, and HA as *grayish* to *black*. Histomorphometry results **(b, c)**. Defect bridging **(b)** and new bone formation **(c)** decrease significantly in ADMS microarchitecture with the increase in minimal constriction from 500 to 1100 μm, as evident by the Jonckheere–Terpstra test results. The lattice microarchitecture yielded the highest values but not significantly higher than with ADMS 500 μm or ADMS 800 μm scaffolds. Values are displayed as box plots ranging from the 25th (lower quartile) to the 75th (upper quartile) percentile. The median is displayed as a *solid black line* and *whiskers* extending to the minimum and maximum values. Values outside the range of the box blot are shown as individual points. *p*-Values are provided in the graphs.

One aim of this study was to determine the osteoconductivity and bone regeneration capacity of the three different ADMS-based scaffolds compared with the highly osteoconductive lattice-based scaffold at 4 weeks postoperation. Toluidine Blue-stained ground sections from the middle of each scaffold (Fig. 3a) revealed that bone formation and advancement of mineralized bone tissue into the ADMS-based scaffold from HA varied with minimal constriction in the scaffold. The lattice microarchitecture performed the best under both measures (Fig. 3a); however, it was not significantly better than the ADMS 500 μm. The extent of bone bridging and formation in the defect treated with HA-based scaffolds is illustrated (Fig. 3b, c). Approximately 93.00% ± 8.99%, 82.85% ± 18.48%, and 62.95% ± 19.51% of the middle section was bridged with ADMS 500, 800, and 1100 μm, respectively. In lattice microarchitectures, bridging was 96.85% ± 6.75% complete and significantly higher than that of ADMS 1100 μm.

In the AOI, the percentage of bony regeneration in the middle section was 56.90% ± 15.90%, 49.86% ± 20.90%, and 36.65% ± 18.47% with ADMS 500, 800, and 1100 μm, respectively. In scaffolds with the lattice microarchitecture, the bony regenerated area covered 64.66% ± 15.55% of the AOI and was significantly higher compared with the ADMS 1100 μm. These results suggest that for ADMS microarchitectures in HA, osteoconductivity and bone regeneration decrease with an increase in minimal constriction from 500 to 1100 μm. Notably, ADMS 800 μm built with Ti performed significantly worse for bony bridging and regeneration than that built with HA (Table 2). To better understand this result, we switched our viewpoint from the STL-file embedded minimal constriction in the scaffold to the diameter of the largest sphere that fits into the pore system based on the histology derived from our *in vivo* experiments. For constructs produced in HA, the maximal diameter of the sphere was 1.53 ± 0.12 mm for ADMS 500 μm, 1.51 ± 0.15 mm for ADMS 800 μm, 1.86 ± 0.17 mm for ADMS 1100 μm, and 1.07 ± 0.04 mm for the lattice microarchitecture (Table 1).

Since ADMS 500 μm, ADMS 800 μm, and lattice microarchitecture yielded a significantly higher percentage of defect bridging than ADMS 1100 μm, these results suggest that for high osteoconductivity, the largest sphere that fits into the pore system should not exceed 1.53 mm in diameter (Fig. 3c). For ADMS 800 μm produced in Ti, the maximal sphere diameter was 1.76 ± 0.12 mm and exceeds the maximal diameter for optimal osteoconduction of 1.53 mm. As a result, bony bridging dropped significantly from 82.85 ± 18.48 for ADMS 800 μm in HA to 50.94 ± 26.07 for ADMS 800 μm in Ti and the percentage of bony regenerated area from 65.46 ± 42.20 to 28.53 ± 25.60, respectively (Table 2). These results support our notion that the central determinant of osteoconduction and bone regeneration is the diameter of the largest sphere that fits into the pore system.

### Osteoconductivity, bony regeneration capacity, and mechanical strength of ADMS 500 μm and lattice-based scaffolds in critical-size defects *in vivo*

**FIG. 4.**
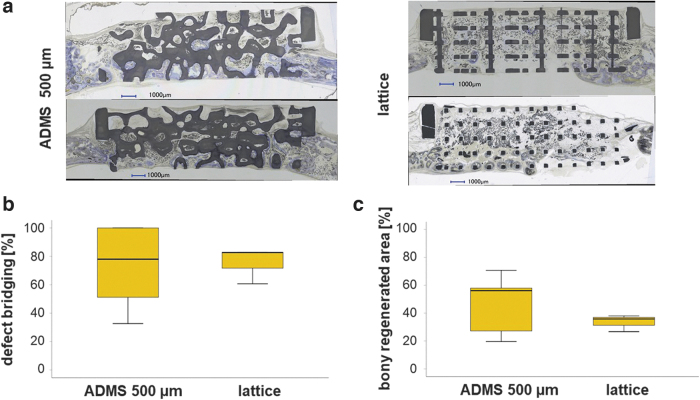
Osteoconduction and bone regeneration in ADMS 500 μm and lattice microarchitecture in a critical-size defect. Two histological sections from the middle of the critical-size defects treated with ADMS 500 μm and a lattice-derived scaffold after 8 weeks of recovery **(a)**. Scale bars in *blue* indicate 1000 μm. Original magnifications were 100-fold. Bone appears as *grayish purple* to *purple* and HA as *grayish* to *black*. Histomorphometry results **(b, c)**. No significant difference could be detected in defect bridging **(b)** and new bone formation **(c)** between ADMS 500 μm and the lattice microarchitectures. Values are displayed as box plots ranging from the 25th (lower quartile) to the 75th (upper quartile) percentile. The median is displayed as a *solid black line* and *whiskers* extending to the minimum and maximum values.

We compared the best-performing ADMS microarchitecture, ADMS 500 μm, and the lattice architecture in a critical-size bone defect model. The scaffolds (Fig. 1d, right scaffold) with the ADMS 500 μm microarchitecture and the lattice architecture were designed to span a 15-mm defect in a rabbit calvarium full-thickness defect model. Healing in terms of defect bridging and bone regeneration was evaluated after 8 weeks. The histology of the middle section (Fig. 4a) revealed that mineralized bone tissue advanced in ADMS 500 μm constructs to span 65.46% ± 29.60% of the defect and 71.67% ± 15.55% in lattice microarchitectures (Fig. 4b). The bony regenerated area was 46.30% ± 21.78% and 33.50% ± 6.00% in ADMS 500 μm constructs and lattice microarchitecture, respectively (Fig. 4c). No significant difference between ADMS 500 μm and lattice constructs was detected in critical-size cranial defects in rabbits over 8 weeks. However, the implant integrity of the scaffolds for critical-size defects was significantly higher with ADMS 500 μm, where five out of the five implants remained 100% intact after the 8-week period in the *in vivo* situation.

The lattice-based scaffolds disintegrated in four out of seven implants, reflecting the much higher mechanical strength of the ADMS-based structure than the lattice architecture (Fig. 2b). The scatter within the lattice structures is more than three times smaller than within the ADMS structures (Fig. 4c) and may indicate improved predictability of osteoconductivity.

## Discussion

TPMS-based structures are promising microarchitectures for bone substitutes because they combine lightweight with mechanical strength. However*, in vivo* results using TPMS microarchitectures are scarce. In this study, we screened several ADMS-based microarchitectures, distinguished from the TPMS algorithm by an evenly distributed surface orientation and isotropic mechanical properties, for osteoconductivity and bone regeneration in rabbit calvarial defect models. In the noncritical-size defect model, an increase in minimal constriction in the ADMS structure was associated with an increase in maximum pore size and a significant decrease in osteoconductivity in terms of defect bridging and bone regeneration. The level of both measurements was not significantly different between ADMS 500 μm and the highly osteoconductive lattice architecture.

However, the compression strength of the latter was significantly lower compared with all the tested ADMS structures. The superior mechanical stability of the ADMS 500 μm was mirrored by the high failure rate of the lattice architecture in critical-size defects. However, both structures performed equally well in the critical-size cranial defect model in terms of osteoconductivity and bony regeneration.

Lightweight microarchitectures depending on the minimal surface algorithm are optimal for use as bone substitutes. Initially, three ADMS microarchitectures were chosen based on the defined minimal constrictions of 500, 800, and 1100 μm. Our results suggest that the central determinant of osteoconduction and bone regeneration is the diameter of the largest sphere that fits into the pore system. If the diameter is ≤1.53 mm, as found in ADMS 500 μm, ADMS 800 μm, and the lattice microarchitecture (Table 1), osteoconduction is significantly higher than with ADMS 1100 μm with a 1.83 mm as the largest sphere diameter fitting into the interconnected pore system. Currently, scaffolds based on TPMS–microarchitectures with pores up to 1.00 mm,^42^ gyroids with 0.43 mm constrictions, and 0.81 mm pore diameter have been tested.^32^ Suggestions for an optimal pore size range for bone regeneration could not be drawn from these studies. Published literature on bone regeneration with scaffolds containing pores >1.00 mm is scarce.

In drill hole defects in sheep treated with ceramic scaffolds with randomly distributed pores of 0.15–1.22 mm in diameter, the pore size did not affect bone healing.^43^ With uniform pore diameters in a rabbit defect model, we demonstrated that the optimal pore diameter for osteoconduction and bone regeneration was between 0.7 and 1.2 mm. However, diameters of 1.5–1.7 mm disabled osteoconduction and bone regeneration substantially.^41^ This shift in the optimal pore diameter from 1.20 mm for an ordered pore-based microarchitecture to 1.53 mm for ADMS microarchitectures might be because, in the first case, all pores are 1.20 mm in diameter. In contrast, only a single pore in each section was 1.53 mm in diameter in ADMS microarchitectures.

Surprisingly, HA-based ADMS 800 μm performed significantly better than the corresponding scaffold made from titanium (Table 2). This appears to be because production-dependent minor changes in wall thickness influence the diameter of the maximum sphere fitting into the microarchitecture. A material-dependent influence appears unlikely since titanium and calcium phosphate-based microarchitectures perform equally well.^40^ It also suggests that the microporosity (interconnected pores in the micrometer range within the sintered material^38^) of 15% found in these HA-based scaffolds^38^ versus a microporosity of 0% in titanium scaffolds does not affect osteoconductivity. For the ADMS 800 μm scaffolds made from titanium, the diameter of the maximum sphere fitting into the microarchitecture was 1.76 mm, and is significantly higher than the 1.51 mm found in the HA-based scaffold (Table 2). This result further confines the maximum pore diameter value in ADMS microarchitectures for optimal osteoconduction and bony regeneration to ∼1.53–1.76 mm.

Discrepancies between the STL file and printed microarchitecture owing to the production methodology should be considered. Moreover, in the ADMS- and TPMS algorithm used to design the bone substitute microarchitecture, the maximum pore diameter should be set at 1.53 mm to facilitate optimized osteoconduction and defect healing. The minimal constriction can be set to 0.50 mm since ADMS 500 and 800 μm performed equally well if made from HA (Fig. 3). In particular, for powder-bed-based 3D-printing methodologies, the geometric constrictions should be as wide as possible to enable the efficient release of powder entrapped in the structure. As ADMS comprises two continuous but independent pore systems, powder removal is possible, and in our ground sections from ADMS 800 μm in titanium, we did not find a single pore filled with powder. We did not evaluate this possibility for the ADMS 500 μm design.

ADMS-based scaffolds showed higher mechanical strength than the lattice microarchitecture (Fig. 2) quantitatively in mechanical compression tests and qualitatively in critical-size defects in *in vivo* situations, which could partially be explained by the higher porosity according to the Gibson–Ashby relation.^44^ These results reflect not only different porosities but also different strength distributions in such architectures because ADMS, like TPMS structures, are doubly curved at each point such that the mean curvature is zero at every point. Although lattice architectures can be adjusted to mechanical needs,^45^ smooth and continuous structures such as primitive-TPMSs are less likely to cause stress concentration, yielding an overall higher scaffold strength.^46^ Moreover, they are optimized for low weight and require less material.

For gyroid-TPMS^47^ the mechanical properties reportedly corresponded well to the material density.^48^ Therefore, as seen in ADMS 500 μm, the least porous scaffold should exhibit the highest strength. To determine the differences between the three ADMS structures presented in this study, additional studies with structures optimized for mechanical testing are required and will be evaluated in the future. Owing to the brittle nature of ceramic materials, such studies should be performed using titanium-based scaffolds.

Osteoconduction depends on a currently undefined communication guided by the scaffold between defect margins.^5,41^ Therefore, it is no surprise that lattice microarchitectures that are highly transparent in terms of the percentage of material-free area in the projection through the entire scaffold show high osteoconductivity. However, the ADMS 500 μm and ADMS 800 μm microarchitectures with zero transparency are statistically indistinguishable from the osteoconductivity of the lattice microarchitectures (Figs. 3 and 4). Transparency is not a critical limiting factor for osteoconductivity and has been reported for pore-based microarchitectures.^41^ Moreover, in lattice architectures, the straight and shortest possible paths for bone ingrowth aimed at defect bridging are unobstructed, and all possible routes are interconnected. However, the two paths defined by the two-pore systems are not interconnected in ADMS structures and are neither straight nor short but instead are slightly curved. Therefore, guiding and communication for osteoconduction can occur efficiently in curved microarchitectures and independently in the two separated channel systems.

One could speculate that for more extended defects, straight and short bone in-growth patterns, as facilitated in lattice architectures, are superior to curved ones, as found in ADMS architectures because the maximum reach for a guiding cue might be limited. Therefore, additional studies and animal model systems are required to provide a definitive answer to this question.

## Conclusion

Lightweight, high-strength ADMS microarchitectures can be designed to be highly osteoconductive and, therefore, well suited for the microarchitecture of bone substitutes. The key design criterion is the 2D maximal sphere diameter fitting into the microarchitecture. If high osteoconductivity and optimal bone regeneration are desired, its value should not exceed 1.53 mm. In combination with a minimal constriction, a 500 μm ADMS microarchitecture appears well suited for bone substitutes used in clinical settings for load-bearing and nonload-bearing applications.

## Institutional Review Board Statement

The procedure involving animals was evaluated and accepted by the local authorities (Veterinäramt Zürich) and provided with the license 065/2018.

## Data Availability Statement

The raw/processed data required to reproduce these findings cannot be shared at this time as the data also form part of additional ongoing studies.
